# Protective Effects of Thalidomide on High-Glucose-Induced Podocyte Injury through *In Vitro* Modulation of Macrophage M1/M2 Differentiation

**DOI:** 10.1155/2020/8263598

**Published:** 2020-08-27

**Authors:** Hui Liao, Yuanping Li, Xilan Zhang, Xiaoyun Zhao, Dan Zheng, Dayue Shen, Rongshan Li

**Affiliations:** Department of Pharmacy, Shanxi Provincial People's Hospital of Shanxi Medical University, Taiyuan 030012, China

## Abstract

*Objective*. It has been shown that podocyte injury represents an important pathological basis that contributes to proteinuria and eventually leads to kidney failure. High glucose (HG) activates macrophage polarization, further exacerbating HG-induced podocyte injury. Our previous study on diabetic nephropathy rats indicated that thalidomide (Tha) has renoprotective properties. The present study explored the effects of Tha on mRNA and protein expressions of inducible nitric oxide synthase (iNOS), tumor necrosis factor- (TNF-) *α*, mannose receptor (CD206), and arginase- (Arg-) 1 in HG-activated macrophages. iNOS and TNF-*α* are established as markers of classically activated macrophage (M1). CD206 and Arg-1 are regarded as markers of alternatively activated macrophages (M2). During the experiment, the supernatants of (HG)-treated and (Tha)-treated macrophages, designated as (HG) MS and (Tha) MS, were simultaneously collected and processed. TNF-*α* and interleukin- (IL-) 1*β* levels as well as protein expressions of nephrin and podocin in HG, (HG) MS, and (Tha) MS-cultured podocytes were evaluated. The results showed that compared to the 11.1 mM normal glucose (NG), the 33.3 mM HG-cultured RAW 264.7 cells exhibited upregulated iNOS and TNF-*α* mRNAs and protein expressions, and downregulated CD206 and Arg-1 expressions significantly (*p* < 0.05). Tha 200 *μ*g/ml suppressed iNOS and TNF-*α*, and promoted CD206 and Arg-1 expressions significantly compared to the HG group (*p* < 0.05). Furthermore, (HG) MS-treated podocytes showed an increase in TNF-*α* and IL-1*β* levels and a downregulation in nephrin and podocin expression significantly compared to NG-treated and HG-treated podocytes (*p* < 0.05). The (Tha 200 *μ*g/ml) MS group exhibited a decrease in TNF-*α* and IL-1*β* level, and an upregulation in nephrin and podocin expressions significantly compared to the (HG) MS group (*p* < 0.05). Our research confirmed that HG-activated macrophage differentiation aggravates HG-induced podocyte injury *in vitro* and the protective effects of Tha might be related to its actions on TNF-*α* and IL-1*β* levels via its modulation on M1/M2 differentiation.

## 1. Introduction

The 60-year history of thalidomide (Tha) is riddled with tragedy, resilience, and redemption [1]. It started in the late 1950s when Tha was given to pregnant women to relieve morning sickness. It was later (in 1991) confirmed that Tha selectively inhibited the production of human monocyte tumor necrosis factor- (TNF-) *α* in lipopolysaccharide- (LPS-) triggered cells [2].

From then on and for the next two decades, the redemption road was full of hardship until Tha became the first agent to gain approval by the FDA for the treatment of plasma cell myeloma in 2006 [3]. So far, its potential immunomodulatory, anti-inflammatory, and antiangiogenic properties make it a good candidate for the treatment of many diseases such as multiple myeloma, Behçet's syndrome, and inflammatory bowel disease [4–6].

Our preliminary work confirmed that pharmacologically and clinically, Tha had protective effects in diabetic renal injury [7–9]. Diabetic kidney disease (DKD) is considered an immune-mediated disease, with increasing emerging evidence suggesting greater immunological component in its pathophysiology. Infiltration of immune cells, predominantly macrophages, into the diabetic kidney has been reported in a number of both experimental and clinical studies [10]. Macrophage polarization could induce podocyte injury, which is a typical characteristic of DKD [11]. Blocking activated macrophage subtype-derived TNF-*α* could be an important therapeutic approach for the treatment of DKD [12].

In the present study, we firstly explored the effects of Tha on high-glucose- (HG-) induced macrophage polarization. The classically activated macrophage (M1, damaged type) markers including inducible nitric oxide synthase (iNOS), TNF-*α*, and the markers of alternatively activated macrophage (M2, protective type) including mannose receptor (CD206) and arginase- (Arg-) 1 were compared. Secondly, we proceeded to assess the effects of Tha on podocyte injury via macrophage polarization. TNF-*α* and interleukin- (IL-) 1*β* levels and nephrin and podocin protein expressions were compared in podocytes treated with macrophage supernatant.

## 2. Methods

### 2.1. Cell Culture

The conditionally immortalized mouse MPC-5 podocyte cell line was purchased from Fuheng Biology Company (Shanghai, China) and cultured at 33°C in a 10% FBS (Gibco BRL, Gaithersburg, MD, USA) and recombinant interferon- (IFN-) *γ* (G1021, Achieve Perfection, Explore the Unknown, USA) supplemented RPMI-1640 (HyClone, GE Healthcare Life Sciences, Logan, UT, USA) medium. Podocytes were reseeded and cultured at 37°C in an RPMI-1640 medium with 10 mg/ml type-I collagen (BD Bioscience, Bedford, MA, USA) and without IFN-*γ* for 7–15 days to induce differentiation. Then, podocytes were cultured with serum starvation overnight before the stimulation.

The conditionally immortalized mouse RAW 264.7 macrophage cell line was purchased from the Absin Biotechnology Company (Shanghai, China) and cultured per the instructions. RAW 264.7 cells were briefly cultured in a 10% FBS supplemented with RPMI-1640 medium at 37°C in a 5% CO_2_ incubator. The medium was then replaced with a new culture medium the next day. The serum-free RPMI-1640 medium was synchronized for 12 hours before the intervention.

### 2.2. Determination of the HG-Induced Phenotypic Transition of M1 Macrophage [13–15]

RAW 264.7 cells (99 *μ*l, plated at 1 × 10^6^ cells/ml) were stimulated with 25.0 mM, 33.3 mM, and 44.4 mM glucose after 12 hours, 24 hours, and 48 hours, before assessment of nitric oxide (NO) production. The 11.1 mM normal glucose (NG) group was considered as the normal group [13, 14]. LPS-stimulated cells (1 *μ*l, 0.5 *μ*g/ml, Wako Chemicals USA Inc., Richmond, VA, USA) were cultured in a normal medium and considered as the model control [15, 16]. Dimethyl sulfoxide (DMSO) was used as the solvent control of LPS. Nitrite, a stable end-product of NO metabolism, was measured using the Griess reaction.

### 2.3. Effects of Tha and HG on the Viability of Podocytes and Macrophages

Podocytes and macrophages were separately seeded into 96-well plates at a density of 5 × 10^4^ cells/ml and cultured in a 10% FBS RPMI-1640 medium for 24 hours. Following another 24-hour treatment with 11.1 mM glucose, mannitol control, 33.3 mM glucose, and Tha (at 25 *μ*g/ml (Tha25), 50 *μ*g/ml (Tha50), 100 *μ*g/ml (Tha100), and 200 *μ*g/ml (Tha200)) in 33.3 mM glucose, the supernatants were removed, and each well was washed with PBS before the addition of 10% FBS RPMI-1640 medium and 10 *μ*l CCK-8 reagent (Boster Biological Technology, Wuhan, China). Cell viability was determined by measuring the absorbance at 450 nm using a microporous plate reader (Model 550; Bio-Rad Laboratories, Inc., Hercules, CA, USA) after an incubation period of 2 hours at 37°C. The average optical density was determined by examining six wells per group.

### 2.4. Effects of Tha on iNOS, TNF-*α*, Arg-1, and CD206 Protein Expressions in HG-Induced Macrophages (Western Blot)

The treated cells in the 11.1 mM glucose control, the 33.3 mM glucose model, the LPS model control [16], and the Tha50, Tha100, and Tha200 in the 33.3 mM glucose groups were removed from the culture medium after 24 hours and extracted using the RIPA lysis buffer (for 30 minutes) from Solarbio Science & Technology (Beijing, China). Protein concentrations were determined using a BCA Protein Assay Kit from Boster Biological Technology (Wuhan, China). Samples containing 50 *μ*g of protein were run through 12% SDS-PAGE electrophoresis and transferred to the nitrocellulose membranes (Solarbio Science & Technology, Beijing, China). Nonspecific binding was blocked by immersing the membranes into 5% nonfat dried milk and 0.1% (*v*/*v*) Tween-20 in PBS for 3 hours at room temperature. After several consecutive rinses with a washing buffer (0.1% Tween-20 in PBS), the membranes were incubated with primary antibodies against iNOS at 1 : 500 dilution (Catalog No. BA0362, Boster), TNF-*α* (Catalog No. BA0131, Boster) at 1 : 500 dilution, CD206 (Catalog No. A02285-2, Boster) at 1 : 500 dilution, and antibody against Arg-1 (Catalog No. BM4000, Boster) at 1 : 500 dilution overnight at 4°C. The membranes were then washed several times and incubated with the corresponding anti-mouse secondary antibody (Proteintech, Wuhan, China) at room temperature for 3 hours. Subsequently, the analysis was performed on the Quantity One analysis system (Bio-Rad, Hercules, CA, USA). GAPDH was used as an internal loading control at a dilution of 1 : 1000 (Catalog No. A00227-1, Boster).

### 2.5. Effects of Tha on iNOS, TNF-*α*, Arg-1, and CD206 mRNA Expressions in HG-Induced Macrophages (Real-Time Quantitative PCR (qPCR))

Total RNAs were extracted from the 11.1 mM glucose, 33.3 mM glucose, LPS, and Tha-treated cells by TRIzol Reagent (Ambion, USA). An equal amount (1 *μ*g) of RNAs was reverse transcribed using a high-capacity RNA-to-cDNA PCR kit (Takara, Beijing, China). Mouse gene PCR primer sets for TNF-*α*, Arg-1, and CD206 were obtained from SA Biosciences (Germantown, MD). The Power SYBR Green PCR Master Mix (Applied Biosystems) was used with the step-one-plus real-time PCR system (Applied Biosystems). The protocol included denaturing for 15 min at 95°C, then 40 cycles of three-step PCR including denaturing for 15 sec at 95°C, annealing for 30 sec at 58°C, and extension for 30 sec at 72°C, with an additional 15-second detection step at 81°C, followed by a melting profile from 55°C to 95°C at a rate of 0.5°C per 10 sec. 25 ng samples of cDNA were analyzed in quadruplicates in parallel with RPLP1/3 controls. Standard curves (threshold 1 cycle vs. log 2 pg cDNA) were generated from a series of log dilutions of standard cDNAs (reverse transcribed from mRNAs of RAW 264.7 cells in growth medium) from 0.1 pg to 100 ng. Initial quantities of experimental mRNA were then calculated from the standard curves and averaged using the SA Bioscience software. The ratio of the experimental four marker genes to RPLP1/3 mRNA was calculated and normalized to the 11.1 mM glucose control.

### 2.6. Collection of Supernatants from HG- and Tha-Treated Macrophages [17]

RAW 264.7 cells were seeded into six-well plates and cultured in 33.3 mM glucose, Tha50, Tha100, and Tha200 in 33.3 mM glucose, respectively. The supernatants were collected 24 hours later and centrifuged at 1500 *g* for 15 minutes and then labeled as (33.3 mM glucose) MS, (Tha50) MS, (Tha100) MS, and (Tha200) MS. The collected supernatants were filtered with 0.22 *μ*m sterile membranes and used immediately.

### 2.7. Determination of TNF-*α*, IL-1*β*, Nephrin, and Podocin Expressions in Podocytes

Podocytes were separately treated with 11.1 mM glucose, 33.3 mM glucose, (33.3 mM glucose) MS, (Tha50) MS, (Tha100) MS, and (Tha200) MS. Cell supernatants were then harvested after 24 hours and centrifuged at 1500 *g* for 10 minutes at 4°C. TNF-*α* and IL-1*β* levels were determined by an ELISA kit (Catalog No. EK0527 and EK0394, Boster). Absorbance was measured using a microplate reader (Model 550; Bio-Rad Laboratories, Inc.). Each sample was repeatedly tested six times.

The treated podocytes were extracted and collected. Then, protein expressions of nephrin and podocin were determined using the method described in Section 2.4. Antibodies against corresponding proteins, nephrin (Catalog No. A01991, Boster) and podocin (Catalog No. BA1688-2, Boster), were used at 1 : 500 dilution. The procedure was repeated in triplicate for each sample.

### 2.8. Statistical Analysis

The SPSS 19.0 software (IBM, Armonk, NY, USA) was used for statistical analysis. Data were expressed as the mean ± standard error of the mean. Comparisons among groups were conducted by one-way analysis of variance followed by Dunnett's multiple comparisons test for continuous variables. All reported *p* values were two-tailed, and a *p* < 0.05 was considered statistically significant.

## 3. Results

### 3.1. Determination of HG-Induced Phenotypic Transition of M1 Macrophage

**Figure 1:**
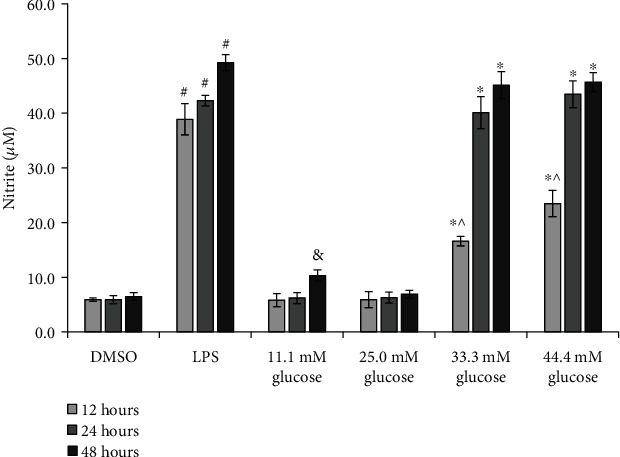
Effects of high glucose on nitric oxide production in RAW 264.7 cells. Values were expressed as the mean ± standard error of the mean (*n* = 6). ^#^*p* < 0.05; LPS versus DMSO after 12, 24, and 48 hours' treatment separately. ^∗^*p* < 0.05; 33.3 and 44.4 mM glucose versus 11.1 mM glucose after 12, 24, and 48 hours' treatment separately. ^^^*p* < 0.05; 33.3 and 44.4 mM glucose versus LPS after 12 hours' treatment. ^&^*p* < 0.05, 11.1 mM glucose versus DMSO after 48 hours' treatment. Abbreviations: LPS: lipopolysaccharide; DMSO: dimethyl sulfoxide, the solvent control of LPS.

The analysis results indicated that the 12-, 24-, and 48-hour LPS-induced NO productions were significantly higher than those of DMSO (the solvent control of LPS) (*p* < 0.001, Figure 1). Taking 0.5 *μ*g/ml LPS as a model control [16], we proceeded to study the concentration of HG.

No significant difference was seen between the 12-, 24-, and 48-hour incubation period NO productions of macrophages in the 25.0 mM glucose group and the 11.1 mM glucose group (all: *p* = NS).

The results also showed that the 12-, 24-, 48-hour incubation period macrophages in the 33.3 mM and 44.4 mM glucose groups had significantly higher nitrite levels as compared to the 11.1 mM glucose group (all: *p* < 0.01). No statistically significant difference in LPS-induced NO production was seen between the 33.3 mM and 44.4 mM glucose groups 24 hours and 48 hours after the treatment (all: *p* = NS).

The comparison between the 11.1 mM glucose and the DMSO groups indicated that NO production was significantly higher in the 11.1 mM glucose 48 hours after the treatment (*p* = 0.005). Meanwhile, no significant difference in NO production was seen between the two groups at 12-hour and 24-hour time points (both: *p* = NS).

Based on above results, the 33.3 mM glucose group and the 24 hours after treatment time point were used as the HG model, while the 11.1 mM glucose group was used as control for both 33.3 mM glucose and LPS.

When compared to 11.1 mM glucose, the LPS-treated, and the 33.3 mM glucose-treated macrophages, the Tha at 25, 50, 100, and 200 *μ*g/ml groups did not exhibit any effects on NO production in 11.1 mM glucose-cultured macrophage. Data did not show.

### 3.2. Effects of Tha and HG on Podocytes and Macrophage Viability

**Figure 2:**
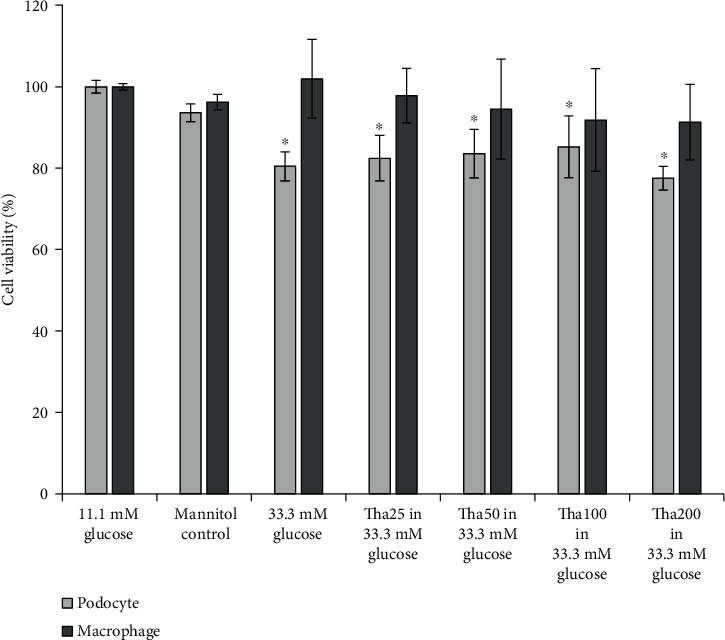
Effects of thalidomide on cell viability of podocyte and macrophage in 33.3 mM glucose. Values were expressed as the mean ± standard error (*n* = 6). ^∗^*p* < 0.05; versus 11.1 mM glucose. Abbreviations: Tha25: 25 *μ*g/ml thalidomide; Tha50: 50 *μ*g/ml thalidomide; Tha100: 100 *μ*g/ml thalidomide; Tha200: 200 *μ*g/ml thalidomide.

Figure 2 shows that the survival rate of podocytes in the 33.3 mM glucose group decreased significantly compared to the 11.1 mM glucose group and also the mannitol control group (both: *p* < 0.001). When compared to the 33.3 mM glucose-treated podocytes, the Tha at 25, 50, 100, and 200 *μ*g/ml groups did not exhibit any protective effects on podocyte cell viability. However, all Tha groups did not show further exacerbation of podocyte death, a trend seen in the 33.3 mM glucose group (all: *p* = NS).

No significant difference in macrophage survival rate was observed between the 33.3 mM glucose, the 11.1 mM glucose, and the mannitol control groups. The results showed that the four tested Tha concentrations had no significant influence on the cell survival rate compared to the 33.3 mM glucose-treated macrophages (all: *p* = NS).

Figure 2 also shows that the survival rate of podocytes in the mannitol control and the 11.1 mM glucose groups did not differ significantly (*p* = NS), neither did the survival rate of macrophages (*p* = NS).

### 3.3. The Effects of Tha on iNOS, TNF-*α*, Arg-1, and CD206 Protein Expressions in 33.3 mM Glucose-Induced Macrophages

**Figure 3:**
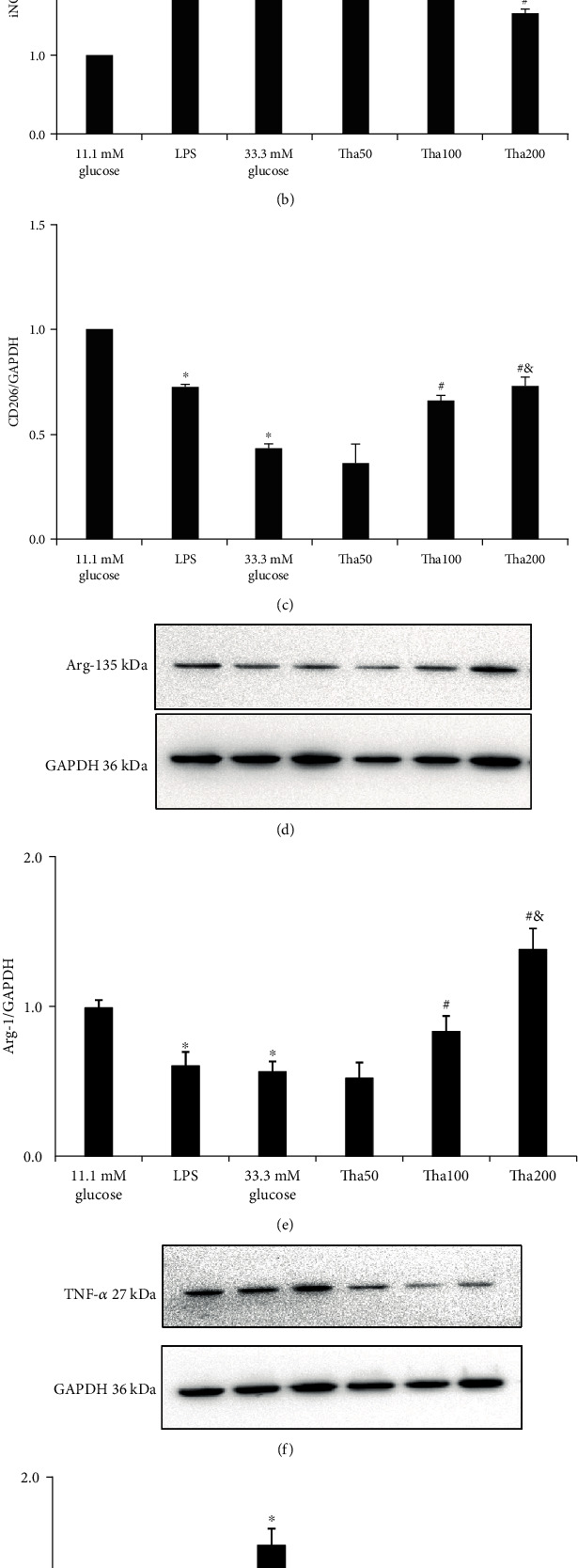
Effects of thalidomide on iNOS, CD206, Arg-1 and TNF-*α* protein expression in 33.3 mM glucose-induced macrophage. (a) iNOS and CD206 protein expressions. (d) Arg-1 protein expression. (f) TNF-*α* protein expression. The results of iNOS, CD206, Arg-1, and TNF-*α* were represented in (b), (c), (e), and (g), respectively. All results were expressed as a ration with respect to control and represented as the mean ± SD in triplicates. ^∗^*p* < 0.05; versus 11.1 mM glucose. ^#^*p* < 0.05; versus 33.3 mM glucose. ^&^*p* < 0.05; versus Tha100. ^^^*p* < 0.05; versus Tha50. Abbreviations: LPS: lipopolysaccharide; Tha50: 50 *μ*g/ml thalidomide in 33.3 mM glucose; Tha100: 100 *μ*g/ml thalidomide in 33.3 mM glucose; Tha200: 200 *μ*g/ml thalidomide in 33.3 mM glucose; iNOS: inducible nitric oxide synthase; CD206: mannose receptor; TNF-*α*: tumor necrosis factor-*α*; Arg-1: arginase-1.

Western blot was used to determine the effects of Tha on iNOS, TNF-*α*, Arg-1, and CD206 protein expressions.  Figure 3 shows that compared to the 11.1 mM glucose group, both the LPS and 33.3 mM glucose-cultured RAW 264.7 cell groups exhibited a significant increase in iNOS and TNF-*α* protein expressions and a significant decrease in Arg-1 and CD206 protein expressions (*p* < 0.05).

The above results further confirmed that the 0.5 *μ*g/ml LPS, as a model control [16], induced M1 polarization but decreased M2 polarization. There were no significant differences in iNOS, TNF-*α*, CD206, and Arg-1 expressions between the LPS model and the 33.3 mM glucose model (all: *p* = NS).

The results of the four different Tha concentrations indicated that compared to the 33.3 mM glucose group, the 50 *μ*g/ml Tha concentration significantly suppressed TNF-*α* expression (*p* = 0.001), while Tha with 100 *μ*g/ml concentration showed significant effects on TNF-*α*, CD206, and Arg-1 expressions (all: *p* < 0.05). Additionally, the 200 *μ*g/ml Tha concentration also had significant effects on TNF-*α*, iNOS, CD206, and Arg-1 expressions (all: *p* < 0.05).

The between different Tha concentrations analysis indicated that the 200 *μ*g/ml Tha concentration showed not only significant effects on CD206 and Arg-1 compared to the 100 *μ*g/ml concentration (*p* = 0.027 and *p* = 0.007, respectively) but also showed significant effects on TNF-*α* compared to the 50 *μ*g/ml concentration (*p* = 0.047).

### 3.4. Effects of Tha on iNOS, TNF-*α*, Arg-1, and CD206 mRNA Expressions in 33.3 mM HG-Induced Macrophages

qPCR was used to determine the effects of Tha on iNOS, TNF-*α*, Arg-1, and CD206 mRNA expressions. Compared to the 11.1 mM glucose group, both the LPS and the 33.3 mM glucose groups showed a significant increase in iNOS and TNF-*α* mRNA expressions and a significant decrease in Arg-1 as well as CD206 mRNA expressions (all: *p* < 0.05).

The 200 *μ*g/ml Tha group exhibited significant effects on iNOS, TNF-*α*, CD206, and Arg-1 expressions compared to the 33.3 mM glucose group (all: *p* < 0.05). Additionally, the 100 *μ*g/ml Tha group also showed significant effects on iNOS, TNF-*α*, and Arg-1 expressions compared to the 33.3 mM glucose group (all: *p* < 0.05). The analysis involving the 50 *μ*g/ml Tha group indicated that 50 *μ*g/ml Tha significantly decreased TNF-*α* expression (*p* = 0.001).

**Figure 4:**
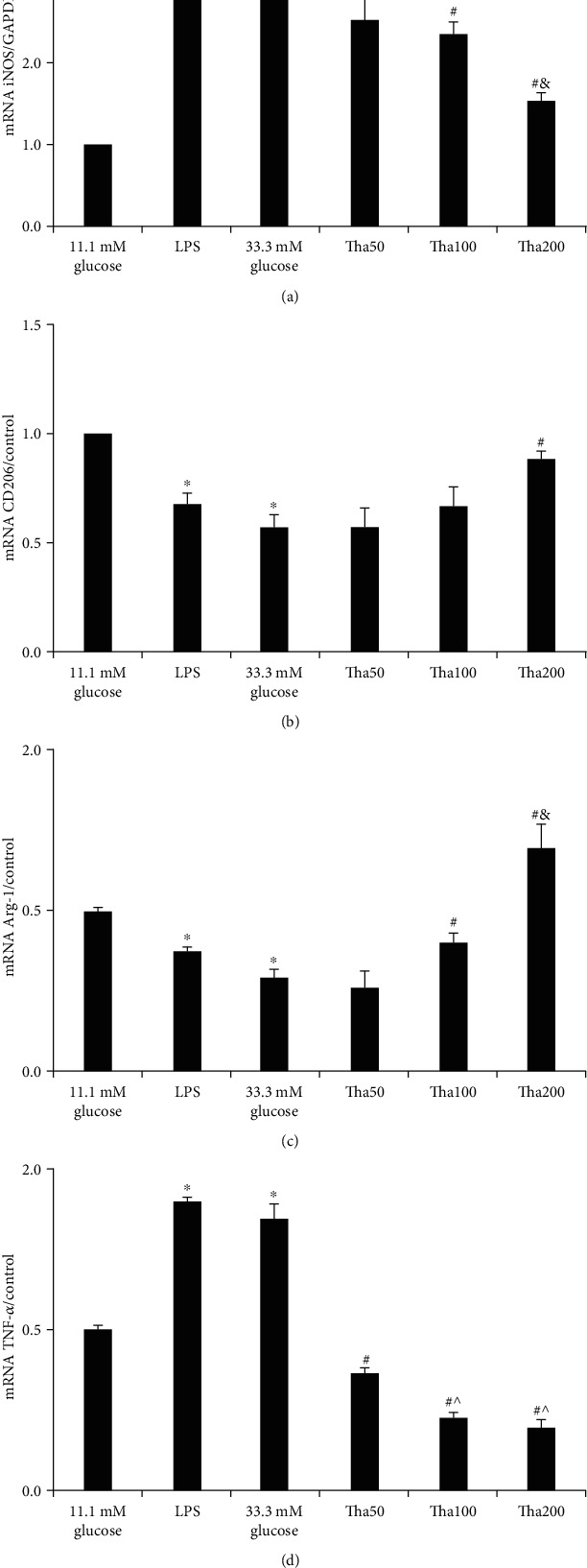
Effects of thalidomide on iNOS, CD206, Arg-1, and TNF-*α* mRNA expressions in 33.3 mM glucose-induced macrophage. (a) iNOS mRNA expression. (b) CD206 mRNA expression. (c) CD206 mRNA expression. (d) TNF-*α* mRNA expression.All the results were represented as the mean ± SD in triplicates ^∗^*p* < 0.05; versus 11.1 mM glucose. ^#^*p* < 0.05; versus 33.3 mM glucose. ^&^*p* < 0.05; versus Tha100. ^^^*p* < 0.05; versus Tha50. Abbreviations: LPS: lipopolysaccharide; Tha50: 50 *μ*g/ml thalidomide in 33.3 mM glucose; Tha100: 100 *μ*g/ml thalidomide in 33.3 mM glucose; Tha200: 200 *μ*g/ml thalidomide in 33.3 mM glucose; iNOS: inducible nitric oxide synthase; CD206: mannose receptor; TNF-*α*: tumor necrosis factor-*α*; Arg-1: arginase-1.

Tha 200 *μ*g/ml concentration showed significant effects on iNOS and Arg-1 compared to Tha 100 *μ*g/ml (*p* = 0.003 and *p* = 0.011). Tha at both 100 *μ*g/ml and 200 *μ*g/ml concentrations had significant effects on TNF-*α* compared to Tha 50 *μ*g/ml concentration (*p* = 0.002 and *p* = 0.001). The above results are shown in Figure 4.

### 3.5. Determination of TNF-*α* and IL-1*β* Levels in Podocytes

**Figure 5:**
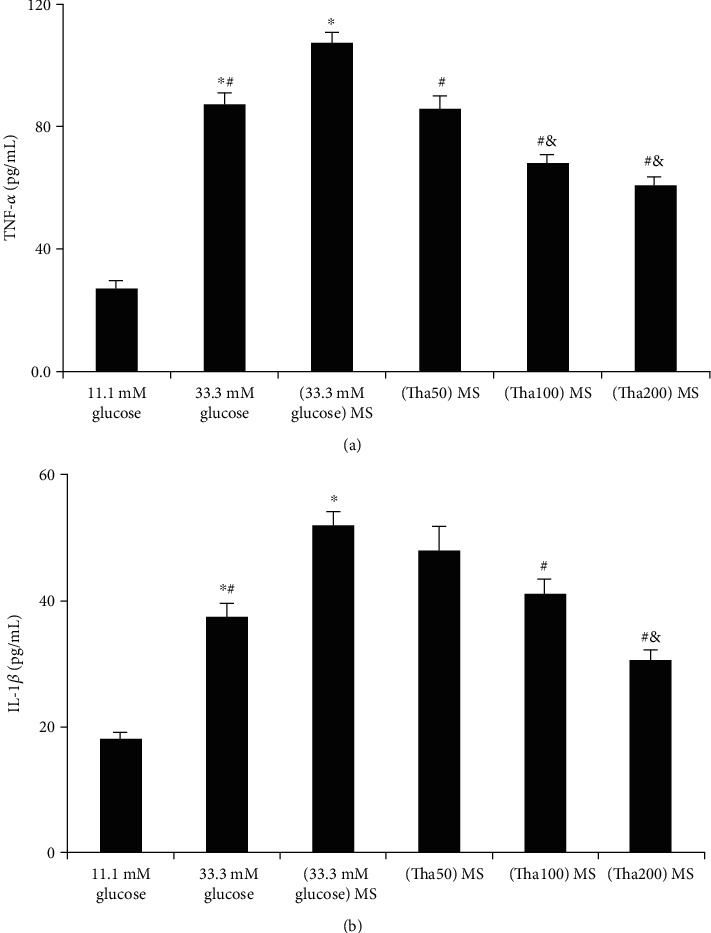
Effects of thalidomide on TNF-*α* and IL-1*β* level in podocyte. (a) TNF-*α* level. (b) IL-1*β* level. Values were expressed as the mean ± standard error of the mean (*n* = 6). ^∗^*p* < 0.05; versus 11.1 mM glucose. ^#^*p* < 0.05; versus (33.3 mM glucose) MS. ^&^*p* < 0.05; versus 33.3 mM glucose. Abbreviations: (33.3 mM glucose) MS: the supernatant from 33.3 mM glucose-treated macrophage; (Tha50) MS: supernatant from 50 *μ*g/ml thalidomide and 33.3 mM glucose-treated macrophage; (Tha100) MS: supernatant from 100 *μ*g/ml thalidomide and 33.3 mM glucose-treated macrophage; (Tha200) MS: supernatant from 200 *μ*g/ml thalidomide and 33.3 mM glucose-treated macrophage.

We can see from Figure 5 that compared to the 11.1 mM glucose group, TNF-*α* levels increased significantly after 33.3 mM glucose stimulation ((76.9 ± 1.6) pg/ml *vs.* (27.0 ± 2.5) pg/ml, *p* < 0.001). Additionally, the (33.3 mM glucose) MS group showed significant promotion of TNF-*α* levels in podocytes ((107.5 ± 3.5) pg/ml) compared to the 33.3 mM glucose group (*p* < 0.001).

The (Tha50) MS, (Tha100) MS, and (Tha200) MS groups showed a significant decrease in TNF-*α* levels compared to that of the (33.3 mM glucose) MS group (all: *p* < 0.001). TNF-*α* levels of both the (Tha100) MS and (Tha200) MS groups were significantly lower than those of the 33.3 mM glucose group ((67.9 ± 3.1) pg/ml and (61.0 ± 2.5) pg/ml *vs.* (76.9 ± 1.6) pg/ml, both: *p* < 0.01).

The comparison in IL-1*β* levels between the 33.3 mM glucose group ((37.5 ± 2.1) pg/ml) and the 11.1 mM glucose group ((18.0 ± 1.2) pg/ml) indicated a significant difference between the two groups (*p* < 0.001). The results also showed that IL-1*β* was significantly promoted in the (33.3 mM glucose) MS group ((52.0 ± 2.2) pg/ml) compared to the 33.3 mM glucose group (*p* < 0.001).

Additionally, IL-1*β* levels were significantly lower in the (Tha100) MS and (Tha200) MS groups as compared to the (33.3 mM glucose) MS group (both: *p* < 0.001), with those levels being significantly decreased in the (Tha200) MS group compared to the 33.3 mM glucose group (*p* = 0.003).

### 3.6. Determination of Nephrin and Podocin Protein Expressions in Podocytes

**Figure 6:**
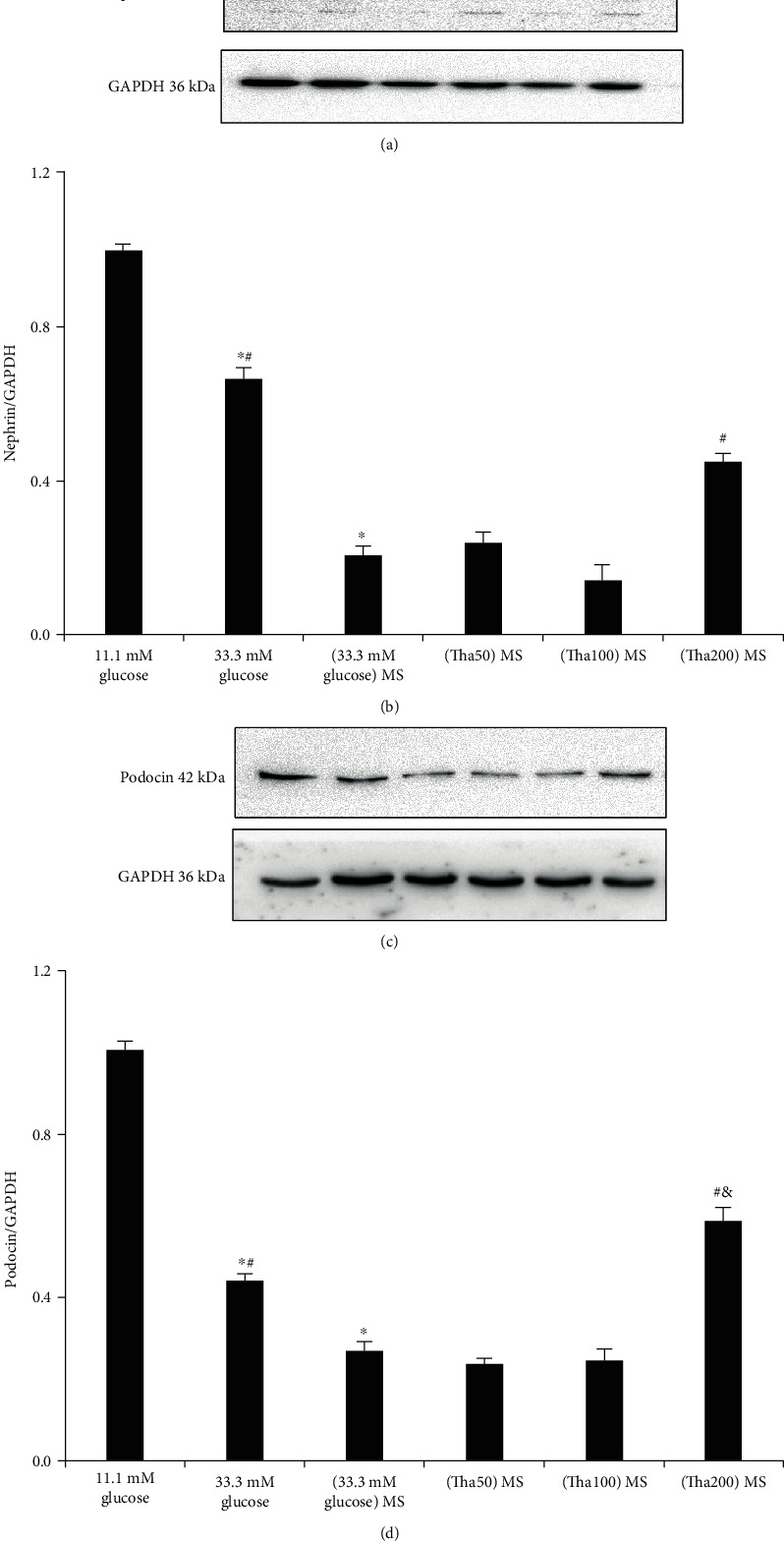
Effects of thalidomide on nephrin and podocin protein expressions in podocyte. (a) Nephrin expression. (c) Podocin expression. The results of nephrin and podocin were represented in (b) and (d). ^∗^*p* < 0.05; versus 11.1 mM glucose. ^#^*p* < 0.05; versus (33.3 mM glucose) MS. ^&^*p* < 0.05; versus 33.3 mM glucose. Abbreviations: (33.3 mM glucose) MS: the supernatant from 33.3 mM glucose-treated macrophage. (Tha50) MS: supernatant from 50 *μ*g/ml thalidomide and 33.3 mM glucose-treated macrophage; (Tha100) MS: supernatant from 100 *μ*g/ml thalidomide and 33.3 mM glucose-treated macrophage; (Tha200) MS: supernatant from 200 *μ*g/ml thalidomide and 33.3 mM glucose-treated macrophage.

Both nephrin and podocin expressions were significantly lower in the 33.3 mM glucose group than in the 11.1 mM glucose group (Figure 6, both: *p* < 0.01). The results also showed a further decrease in nephrin and podocin expressions in the (33.3 mM glucose) MS group as compared to the 33.3 mM glucose group (both: *p* < 0.01).

The Tha-related results indicated a significant increase in nephrin and podocin expressions in the (Tha200) MS group as compared to the (33.3 mM glucose) MS group (both: *p* < 0.001) and a significant increase in podocin expressions when compared to the 33.3 mM glucose group (*p* = 0.006).

## 4. Discussion

During the past 30 years, Tha and its analogs could be alternatively used in the treatment of neurological and renal diseases such as dyskinesia [18], Alzheimer's disease [19], lupus nephritis [20], and DKD [9] due to its immunomodulatory properties. It is reported that Alzheimer's disease is progressed by activated microglia, the resident brain macrophage, and release inflammatory mediators such as TNF-*α* [21]. A study demonstrates that human umbilical cord-derived mesenchymal stem cells ameliorated lupus nephritis by preventing podocyte injury possibly through reducing macrophage infiltration and polarizing macrophage into an anti-inflammatory phenotype [22].

Our preliminary work on streptozotocin-induced rats showed that Tha suppressed the inflammatory and fibrotic processes in diabetic renal injury [8]. These effects were partly mediated by the activation of AMPK*α* and inhibition of the NF-*κ*B/MCP-1 and TGF-*β*1/Smad signaling pathways [7]. The renoprotective effects of Tha were further confirmed clinically [9].

DKD is one of the major complications of diabetes mellitus and is currently the most common cause of end-stage renal disease in China [23]. Recent growing evidence hinted at the participation of immunologic and inflammatory mechanisms in the development and progression of DKD [24]. Macrophage polarization plays a pivotal role in the process of inflammation, a common occurrence in DKD. Therefore, interventions during the M1 and M2 macrophage polarization processes might be a novel therapeutic strategy for DKD [25].

Previous reports have shown that the M1 phenotype can be induced by LPS [16]. The inhibition of iNOS in LPS-induced macrophages may prove to be an important target for the anti-inflammatory effects of Tha [26]. LPS was used in our previous study to induce iNOS [15] and was considered as the model control in the present study.

As a TNF-*α* inhibitor [27], Tha had downregulatory effects on 33.3 mM glucose-induced iNOS and TNF-*α* mRNA and protein expressions in our present study. Both iNOS and TNF-*α* have been regarded as markers of the M1 subtype [28, 29]. Our results also showed that treatment with 33.3 mM glucose significantly downregulated Arg-1 and CD206 protein and mRNA expressions in macrophages, an effect that was reversed with Tha treatment. Interestingly, many studies have established that the upregulation of CD206 and Arg-1 is an important indicator of M2 polarization [30, 31]. This is to our knowledge the first study to explore the effects of Tha on markers of the M2 subtype.

A previous study revealed that activated macrophage played a crucial role in the injury of podocytes located in the outer layer of the filtration barrier. Such injury plays an important role in the inflammatory processes of DKD [11]. We could see that the survival rate of podocytes decreased significantly after treatment with 33.3 mM glucose. Tha neither alleviated nor aggravated the mortality rate of 33.3 mM glucose-cultured podocytes.

According to the present study, two kinds of cells are involved in the inflammatory process: the bone marrow-derived leukocytes, including neutrophils and macrophages, which are firstly activated, and the renal cells such as mesangial cells and podocytes [32]. We hypothesized that Tha has protective impacts on HG-induced podocyte injury via modulation of M1/M2 differentiation. To assess this hypothesis, we used supernatants from 33.3 mM glucose-treated and Tha-treated macrophages to treat podocytes (*in vitro*).

Podocin and nephrin are podocyte-specific markers, and a decrease in their expressions is indicative of podocyte damage [33]. Our results showed that podocin and nephrin protein expressions in the (33.3 mM glucose) MS-cultured podocytes were significantly lower than those in the 33.3 mM glucose-cultured podocytes. This confirmed that HG-induced macrophage polarization aggravated HG-induced podocyte injury. On the other hand, the increase in podocin and nephrin expressions in the (Tha200) MS group proved that the protective effects of Tha might be related to the used supernatant.

Another study demonstrated that activated macrophages could induce podocyte injury via a TNF-*α*-JNK/p38-dependent mechanism [34]. Many inflammatory factors, such as TNF-*α*, IL-1*β*, IL-6, and IL-17, took part in the inflammation process and contributed to podocyte injury [35]. Our research demonstrated that the increase in TNF-*α* and IL-1*β* expressions played an important role in the podocyte injury process *in vitro*. The decreasing effect of Tha on TNF-*α* and IL-1*β* levels might explain the protective effects of Tha on podocyte injury via the macrophage supernatant.

It was previously revealed that vitamin D and calcineurin inhibitor prevented podocyte injury via regulation of macrophage M1/M2 phenotype in diabetic nephropathy rats [36, 37]. The connection between macrophage phenotype and its relationship with renal function and histological changes in human DKD has been extensively explored. It was demonstrated that there is a positive correlation between the M1/M2 differentiation state and the progress of DKD [38].

Metformin showed its podocyte-protective capacity in type 2 diabetic patients, and the underlying mechanisms might be partly attributable to its effects on the M1 polarization-related MIF-CD74 axis [39, 40]. Further exploratory studies on the protective effects of Tha on podocyte via modulation of the balance between the M1/M2 phenotype should be conducted in DKD animal models. The balance between M1/M2 on different progress of DKD models, such as early stages and late chronic tissue damage, will be further compared in our future researches.

Many studies suggested that DKD podocyte injury is induced by the association of multiple factors, including inflammatory reaction, oxidative stress, TGF-*β*1 induction, renin angiotensin aldosterone system activation, and AGEs accumulation [41]. At present study, we just focused only on NO, TNF-*α*, and IL-1*β* as proinflammatory cytokines produced by activated macrophage. The precise mechanism of macrophage-mediated podocyte injury induced by HG, and how podocytes themselves involve in the pathogenesis of DKD should be demonstrated in our future study.

From the perspective of drug toxicity, our research did not indicate significant Tha toxicity on used macrophages and podocytes in 33.3 mM glucose. However, we should still focus on daily dose-dependent toxicity and cumulative dose-dependent toxicity of Tha in animal researches [42–44]. A clinical phase II trial of Tha in patients with metastatic renal cell carcinoma was performed; the results showed that low doses of Tha resulted in manageable toxicity, better response rates, and progression-free survival [43]. However, high doses of Tha are not recommended [42], and reports have shown that a cumulative dose greater than 20 g represented a risk factor [44].

As a therapeutic alternative to traditional anti-TNF-*α* compounds, we should pay more attention to its dose-related side effects, with the most notable being peripheral neuropathy [45]. Many analogs of Tha, such as lenalidomide and pomalidomide, were clinically studied for the treatment of relapsed or refractory multiple myeloma [46, 47]. Simultaneously, the safety parameters of these analogs were closely monitored to achieve maximum clinical benefit [46, 47].

Summary of our results is as follows: (1) Macrophage M1/M2 polarization could be induced by 33.3 mM glucose, and Tha exhibited modulatory effects on the M1/M2 phenotype. (2) 33.3 mM glucose decreased nephrin and podocin expressions in podocytes *in vitro*. 33.3 mM glucose-induced macrophage M1/M2 polarization further exacerbated the decrease in nephrin and podocin expressions. (3) Tha displayed indirect protective effects on podocyte injury through modulation of macrophage M1/M2 differentiation-related activities. (4) Tha-related side effects should always be the center of attention during the experiment.

## Data Availability

The data used to support the findings of this study are available from the corresponding author upon request.
